# Physicians’ use and perceptions of genetic testing for rare diseases in China: a nationwide cross-sectional study

**DOI:** 10.1186/s13023-023-02847-7

**Published:** 2023-08-10

**Authors:** Weida Liu, Peng Liu, Dan Guo, Ye Jin, Kun Zhao, Jiayin Zheng, Kexin Li, Linkang Li, Shuyang Zhang

**Affiliations:** 1 Medical Research Center, State Key Laboratory of Complex Severe and Rare Diseases, Peking Union Medical College Hospital, Chinese Academy of Medical Sciences and Peking Union Medical College, Beijing, 100730 China; 2 Clinical Biobank, Peking Union Medical College Hospital, Chinese Academy of Medical Sciences & Peking Union Medical College, Beijing, 100730 China; 3 Vanke School of Public Health, Institute for Healthy China, Tsinghua University, Tsinghua University, Beijing, China; 4 China Alliance for Rare Diseases, Beijing, China; 5 Department of Cardiology, Peking Union Medical College Hospital, Chinese Academy of Medical Sciences & Peking Union Medical College, Beijing, 100730 China

**Keywords:** Genetic testing, Rare diseases, Accessibility, Utilization

## Abstract

**Background:**

Genetic testing can facilitate the diagnosis and subsequent therapeutic management of rare diseases. However, there is a lack of data on the use of genetic testing for rare diseases. This study aims to describe the utilization rate and troubles encountered by clinicians in treating rare diseases with genetic testing.

**Methods:**

A cross-sectional electronic questionnaire survey was conducted between June and October 2022 among the medical staff from the hospitals covering all provinces, municipalities, and autonomous regions of China. The survey on genetic testing focused on whether genetic testing was used in the diagnosis and treatment of rare diseases, the specific methods of genetic testing, and the problems encountered when using genetic testing.

**Results:**

A total of 20,132 physicians who had treated rare diseases were included, of whom 35.5% were from the central region, 36.7% were from the eastern region, and 27.8% were from the western region. The total utilization rate of genetic testing for rare diseases was 76.0% (95%CI: 75.4–76.6). The use of genetic testing was highest in the Eastern region (79.2% [95% CI: 78.3–80.1]), followed by the Central (75.9% [95% CI: 74.9–76.9]) and Western regions (71.9% [95% CI: 70.7–73.1]). More than 90% (94.1% [95%CI: 93.4–94.8]) of pediatricians had used genetic testing to treat rare diseases, with surgeons having the lowest use of genetic testing (58.3% [95% CI: 56.6–60.0]). Physicians’ departments and education levels affect the use of genetic testing. Most physicians have used a variety of genetic tests in the management of rare diseases, the most popular methods were “Whole-exome sequencing (Proband)” and “Whole-exome sequencing (families of three or more)”. Doctors have encountered many problems with the use of genetic testing in the diagnosis and treatment of rare diseases, among which the high price was the main concern of medical workers.

**Conclusion:**

Three-quarters of physicians used genetic testing in rare disease practice, and there were regional differences in the use of genetic testing. Recognition of the utilization of genetic testing can help identify patterns of resource utilization in different regions and provide a more comprehensive picture of the epidemiology of rare diseases in jurisdictions.

**Supplementary Information:**

The online version contains supplementary material available at 10.1186/s13023-023-02847-7.

## Background

Rare diseases are a general term for a group of diseases with extremely low incidence and prevalence, and there are more than 7,000 known rare diseases [1] in the world. Despite the low prevalence, patients with rare diseases are not “rare” due to a large number of diseases, and there are approximately 300 million patients with rare diseases worldwide [2] currently. As a global problem [3], statistics show that 80% of rare diseases are caused by genetic factors, 50% develop in childhood and 30% of affected children die within five years of age [2]. Since rare diseases are mostly hereditary and caused by genetic defects, genetic testing has become an important tool [4, 5] for the diagnosis and prevention of rare diseases. A screening result for the infant with suspected monogenic disorders [6] showed that genetic testing accurately diagnosed 57.5% of patients, much higher than the accuracy rate of routine diagnostic methods (13.75%).

Accurate genetic testing facilitates not only the diagnosis of genetic disorders but also the subsequent treatment and management of rare diseases [7, 8]. A previous retrospective study of genetic testing for the diagnosis of critically ill infants showed that 65% of patients reported instant clinical value of the diagnosis, 20% received a diagnosis with strongly favorable effects on disease management, and 30% started palliative care [9, 10]. While the clinical practice has shown that genetic testing is not preferred for the diagnosis of genetic disorders and is only considered when other tests fail to confirm the diagnosis. In addition, genetic testing is expensive and access to genetic testing is unequal [11–13] across geographic regions. A cross-sectional survey of access to genetic testing for patients with ataxia and hereditary spastic paralysis (a rare neurological disease) in 21 EU countries [14] showed that 47.6% (10/21) of the countries had some difficulties in accessing genetic testing (the main problem was financial factors).

Clarifying the use of genetic testing in rare diseases can help rationalize the allocation of healthcare resources and improve the diagnosis and treatment of rare diseases [15]. However, there is a lack of data on the utilization rate of genetic testing for rare diseases, and whether there are differences in the use of genetic testing between regions of different economic levels has not yet been reported [16]. In addition, some studies with small samples of a few dozen cases have shown that doctors encounter many problems such as being " unable to understand the results of genetic test reports”, and “not understand the application scope of genetic testing” [16, 17], but whether these studies are representative of the general problem among doctors is unknown.

Therefore, this study surveyed whether there were differences in the utilization rate and troubles encountered by doctors in the diagnosis and treatment of rare diseases in different regions with different economic levels. The aim of this study is to describe the use of genetic testing for rare diseases among clinicians and to provide a basis for the rational provision and allocation of medical resources for rare diseases in the future.

## Methods

### Study design

This study is a national cross-sectional survey. From June and October 2022, a baseline electronic survey was conducted among medical staff in the China collaboration network [18]. The collaboration network [18] was formed in 2019 by hundreds of hospitals with strong rare disease diagnosis and treatment capabilities and a high number of rare disease cases across China [18, 19], covering all provinces, municipalities, and autonomous regions, taking into account hospitals of different economic levels, with the aim of strengthening the management of rare diseases and improving the diagnosis and treatment of rare diseases.

A total of 21,323 individuals who had been treating rare diseases were included in this study. Six were excluded due to missing key information, resulting in the inclusion of 21,317 healthcare workers. This study aimed to describe the use and trouble of genetic testing among physicians in the diagnosis of rare diseases, so 1,185 individuals from functional or other non-clinical departments were excluded, resulting in the inclusion of 20,132 physicians for analysis.

### Questionnarie and data collection

The structure and items of the questionnaire designing using literature review and expert consultation. The distribution of questionnaires mainly relied on the China Alliance for Rare Diseases. All clinicians could fill in the electronic questionnaire by scanning the two-dimensional code on the official website via their mobile phones or computers, and the unique identifier of each questionnaire was confirmed by the hospital department and the name abbreviation of the physician. And all participating physicians were deidentified to preserve the anonymity of the questionnaire. Any questions that clinicians may have about the questionnaire could be answered by consulting the researcher online.

The contents of the survey included the province, hospital, education, title, length of service, and department in which they worked. This study divided the geographical area into the eastern, central, and western regions according to the provinces where the doctors worked; the education levels were divided into bachelor’s degree or below, master’s degree and doctor’s degree; the professional titles were divided into the resident physician, attending physician, associate chief physician, and chief physician; the department was classified as pediatrics, surgical, non-surgical, diagnosis-related; and the length of service was classified as ≤ 5 years, 6–10 years, 11–20 years, 21–30 years and > 30 years.

The questionnaire on the diagnosis and treatment of rare diseases was mainly concerned with whether there had been any previous experience in diagnosing or treating rare diseases. The definition of rare diseases was mainly derived from the First List of Rare Diseases [20] and rare diseases identified in previous literature or databases. The First List of Rare Diseases was jointly published by five national departments, including the National Health Commission, the Ministry of Science and Technology, the Ministry of Industry, and others, and includes 121 rare diseases.

The section on genetic testing in the questionnaire included whether genetic testing had been used in the diagnosis and treatment of rare diseases, the specific method of genetic testing (“Chromosomal microarray analysis”, “Whole-exome sequencing (Proband)”, “Whole-exome sequencing (families of three or more)”, etc.) and problems with the use of genetic testing (“ Genetic testing is too expensive. “, “Do not understand the application scope of genetic testing.“ “The genetic testing results are too extensive to confirm key test results that aid diagnosis.“ etc.).

### Statistical analysis

All results of this study were analyzed according to subgroups in the central, eastern, and western regions of China. Quantitative data were described by mean and standard deviation, and differences between groups were analyzed by analysis of variance. The qualitative data were described by frequency and percentage, and the differences between groups were compared by the chi-square test. As the main objective of this study was to describe the use of genetic testing by physicians in the diagnosis and treatment of rare diseases, the use of genetic testing was described using 95% confidence intervals (CI). Physician characteristics regarding influences on the use of genetic testing were analyzed using multifactorial logistic regression, with results presented using odds ratios (ORs) and 95% CI. All data were cleaned and analyzed with the use of SAS 9.4 software, and two-sided validation was performed by two people. P < 0.05 was considered statistically significant.

## Results

A total of 20,132 physicians who had treated rare diseases were included, of whom 35.5% were from the central region, 36.7% were from the eastern region, and 27.8% were from the western region. Among the interviewed physicians, 21.9% came from pediatrics, 16.1% from surgical departments, 59.4% from non-surgical departments, and 2.6% from diagnosis-related departments. The junior title accounted for 14.3%, the intermediate title for 35.5%, the associate senior title for 28.3%, and the senior title for 21.9%. Nearly 70% of the respondents had graduate education or above, and more than 80% had worked for more than 5 years. There were significant differences (P < 0.001) in the departments, professional titles, education levels, and length of service of the interviewed doctors in different regions (Table 1).

**Table 1:** Baseline characteristics of the physicians and stratified by region

Variables	Total	Central	Eastern	Western	P value
	(N = 20,132)	(N = 7,146)	(N = 7,385)	(N = 5,601)	
Department					< 0.001
Pediatrics	4417 (21.9)	1664 (23.3)	1661 (22.5)	1092 (19.5)	
Surgical	3246 (16.1)	1058 (14.8)	1108 (15.0)	1080 (19.3)	
Non-surgical	11,967 (59.4)	4235 (59.3)	4422 (59.9)	3310 (59.1)	
Diagnosis-related	502 (2.6)	189 (2.6)	194 (2.6)	119 (2.1)	
Title					< 0.001
Resident physician	2868 (14.3)	938 (13.1)	929 (12.6)	1001 (17.9)	
Attending physician	7154 (35.5)	2644 (37.0)	2509 (34.0)	2001 (35.7)	
Associate chief physician	5699 (28.3)	2052 (28.7)	2133 (28.8)	1514 (27.0)	
Chief physician	4411 (21.9)	1512 (21.2)	1814 (24.6)	1085 (19.4)	
Education					< 0.001
Bachelor’s degree or below	3997 (19.9)	1424 (19.9)	874 (11.8)	1699 (30.3)	
Master’s degree	10,209 (50.7)	3999 (56.0)	3449 (46.7)	2761 (49.3)	
Doctorate/postdoc	5926 (29.4)	1723 (24.1)	3062 (41.5)	1141 (20.4)	
Length of service					< 0.001
≤ 5	3273 (16.3)	1219 (17.1)	1019 (13.8)	1035 (18.5)	
6–10	4889 (24.3)	1728 (24.2)	1729 (23.5)	1432 (25.6)	
11–20	6355 (31.6)	2293 (32.1)	2354 (32.0)	1708 (30.5)	
21–30	4000 (19.9)	1332 (18.7)	1606 (21.8)	1062 (19.0)	
>30	1579 (7.9)	565 (7.9)	659 (8.9)	355 (6.4)	

Notes: Data are expressed as number (%), or p values. There are 36 missing values or outliers in the length of service

**Fig. 1:**
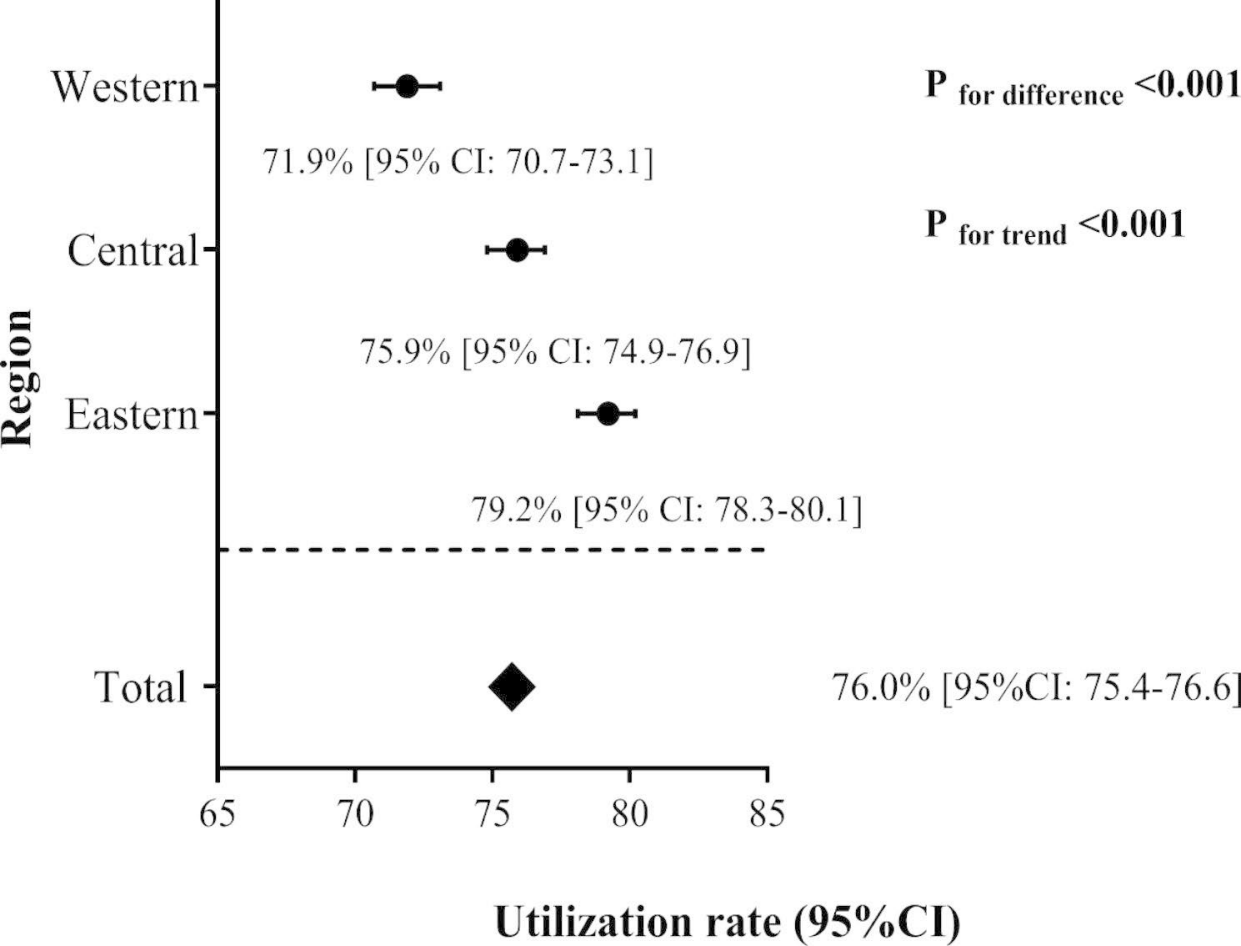
**Utilization rates of genetic testing by physicians in different regions.** The eastern, central and western regions represent different economic levels, with the eastern region being the most developed, followed by the central and western regions. Data are expressed as (column % [95% CI]) or p values

The total utilization rate of genetic testing for rare diseases was 76.0% (95%CI: 75.4–76.6). The use of genetic testing was highest in the Eastern region (79.2% [95% CI: 78.3–80.1]), followed by the Central (75.9% [95% CI: 74.9–76.9]) and Western regions (71.9% [95% CI: 70.7–73.1]) (Fig. 1). More than 90% (94.1% [95%CI: 93.4–94.8]) of pediatricians had used genetic testing to treat rare diseases, and surgeons had the lowest utilization rate of genetic testing (58.3% [95%CI: 56.6–60.0]). There was an upward trend in the use of genetic testing as doctor titles increased (P_trend_ =0.033), but the trend was not statistically significant in the central and eastern regions. The higher the education level of doctors, the higher the utilization rate of genetic testing in the treatment of rare diseases (P_trend_ <0.001), which was consistent across different regions. The use of genetic testing in the diagnosis of rare diseases did not increase with the years of the doctors’ experience (P_trend_ =0.537) (Table 2).

**Table 2:** Rates of use of genetic testing for rare diseases by physicians with different characteristics

Variables	Total	Central	Eastern	Western
	(N = 20,132)	(N = 7,146)	(N = 7,385)	(N = 5,601)
Department				
Pediatrics	94.1% (93.4–94.8)	93.5% (92.3–94.6)	95.9% (95.0-96.9)	92.5% (90.1–94.1)
Surgical	58.3% (56.6–60.0)	57.7% (54.7–60.6)	62.3% (59.7–65.4)	54.6% (51.7–57.6)
Non-surgical	74.4% (73.6–75.2)	73.6% (72.3–75.0)	77.4% (76.1–78.6)	71.4% (69.9–73.0)
Diagnosis-related	68.1% (64.1–72.2)	73.0% (66.7–79.3)	72.7% (66.4–79.0)	52.9% (43.9–61.9)
P value for difference	< 0.001	< 0.001	< 0.001	< 0.001
Title				
Resident physician	75.5% (74.0-77.1)	77.1% (74.4–79.8)	80.8% (78.3–83.4)	69.1% (66.3–72.0)
Attending physician	75.2% (74.2–76.2)	76.0% (74.4–77.6)	77.6% (75.9–79.2)	71.1% (69.1–73.1)
Associate chief physician	76.4% (75.3–77.5)	75.7% (73.8–77.5)	79.2% (77.5–81.0)	73.5% (71.2–75.7)
Chief physician	77.0% (75.8–78.2)	75.1% (73.0-77.3)	80.5% (78.7–82.3)	73.7% (71.1–76.4)
P value for trend	0.033	0.287	0.342	0.007
Education				
Bachelor’s degree or below	66.6% (65.1–68.1)	67.4% (65.0-69.9)	74.0% (71.1–76.9)	62.2% (59.9–64.5)
Master’s degree	75.5% (74.6–76.3)	75.7% (74.3–77.0)	76.8% (75.4–78.2)	73.4% (71.8–75.1)
Doctorate/postdoc	83.2% (82.2–84.1)	83.3% (81.5–85.1)	83.3% (82.0-84.7)	82.7% (80.1–84.8)
P value for trend	< 0.001	< 0.001	< 0.001	< 0.001
Length of service				
≤ 5	76.0% (74.5–77.4)	77.5% (75.2–79.9)	80.3% (77.8–82.7)	69.9% (67.1–72.7)
6–10	76.7% (75.5–77.9)	78.1% (76.1–80.0)	78.8% (76.9–80.8)	72.4% (70.0-74.7)
11–20	75.9% (74.8–76.9)	74.6% (72.8–76.4)	79.8% (78.2–81.4)	72.2% (70.1–74.3)
21–30	75.2% (73.8–76.5)	73.9% (71.5–76.2)	78.3% (76.3–80.3)	72.0% (69.3–74.7)
>30	76.6% (74.5–78.7)	75.6% (72.0-79.1)	78.9% (75.3–81.6)	74.9% (70.4–79.4)
P value for trend	0.537	0.010	0.318	0.126

Notes: Data are expressed as (column % [95% CI]) or p values

After adjusting for multiple factors, the department and education level of the physicians influenced the use of genetic testing. Physicians in pediatrics, non-surgical departments, and diagnostic-related departments had a stronger association with genetic testing in the diagnosis of rare diseases compared to surgical departments. Physicians with master’s degrees and doctoral degrees were more strongly associated with the use of genetic testing in the diagnosis of rare diseases than physicians with undergraduate degrees (Table 3).

**Table 3:** The association of physician characteristics with the use of genetic testing for rare diseases stratified by region

Variables	Total		Central		Eastern		Western	
	OR (95%CI)	P value	OR (95%CI)	P value	OR (95%CI)	P value	OR (95%CI)	P value
Department		< 0.001		< 0.001		< 0.001		< 0.001
Surgical	1.00	−	1.00	−	1.00	−	1.00	−
Pediatrics	13.36(11.54–15.45)		12.46(9.87–15.74)		16.68(12.67–21.97)		11.46(8.84–14.85)	
Non-surgical	2.06(1.89–2.23)		2.02(1.75–2.33)		2.12(1.83–2.44)		2.02(1.75–2.33)	
Diagnosis-related	1.74(1.42–2.14)		2.47(1.74–3.52)		1.68(1.19–2.37)		1.15(0.78–1.7)	
Title		0.443		0.677		0.402		0.679
Resident physician	1.00	−	1.00	−	1.00	−	1.00	−
Attending physician	0.97(0.85–1.11)		0.94(0.75–1.18)		0.95(0.74–1.22)		0.97(0.79–1.2)	
Associate chief physician	1.07(0.91–1.25)		1.04(0.78–1.38)		1.04(0.77–1.4)		1.08(0.82–1.42)	
Chief physician	1.06(0.88–1.27)		0.98(0.7–1.36)		1.15(0.83–1.61)		0.99(0.71–1.38)	
Education		< 0.001		< 0.001		< 0.001		< 0.001
Bachelor’s degree or below	1.00	−	1.00	−	1.00	−	1.00	−
Master’s degree	1.63(1.49–1.78)		1.62(1.39–1.89)		1.17(0.96–1.41)		1.89(1.63–2.18)	
Doctorate/postdoc	3.14(2.83–3.48)		3.35(2.78–4.03)		2.21(1.81–2.69)		3.41(2.8–4.16)	
Length of service		0.238		0.205		0.884		0.074
≤ 5	1.00	−	1.00	−	1.00	−	1.00	−
6–10	1.08(0.95–1.23)		1.10(0.88–1.37)		0.99(0.78–1.27)		1.17(0.94–1.45)	
11–20	1.03(0.89–1.19)		0.92(0.71–1.17)		0.99(0.75–1.29)		1.22(0.95–1.58)	
21–30	1.05(0.88–1.25)		0.98(0.73–1.33)		0.88(0.65–1.2)		1.3(0.95–1.78)	
>30	1.23(0.99–1.52)		1.18(0.82–1.7)		0.87(0.6–1.26)		1.82(1.21–2.73)	

Note: Data are expressed as OR (95% CI) or p values. OR: Odds ratio; CI: confidence interval

**Fig. 2:**
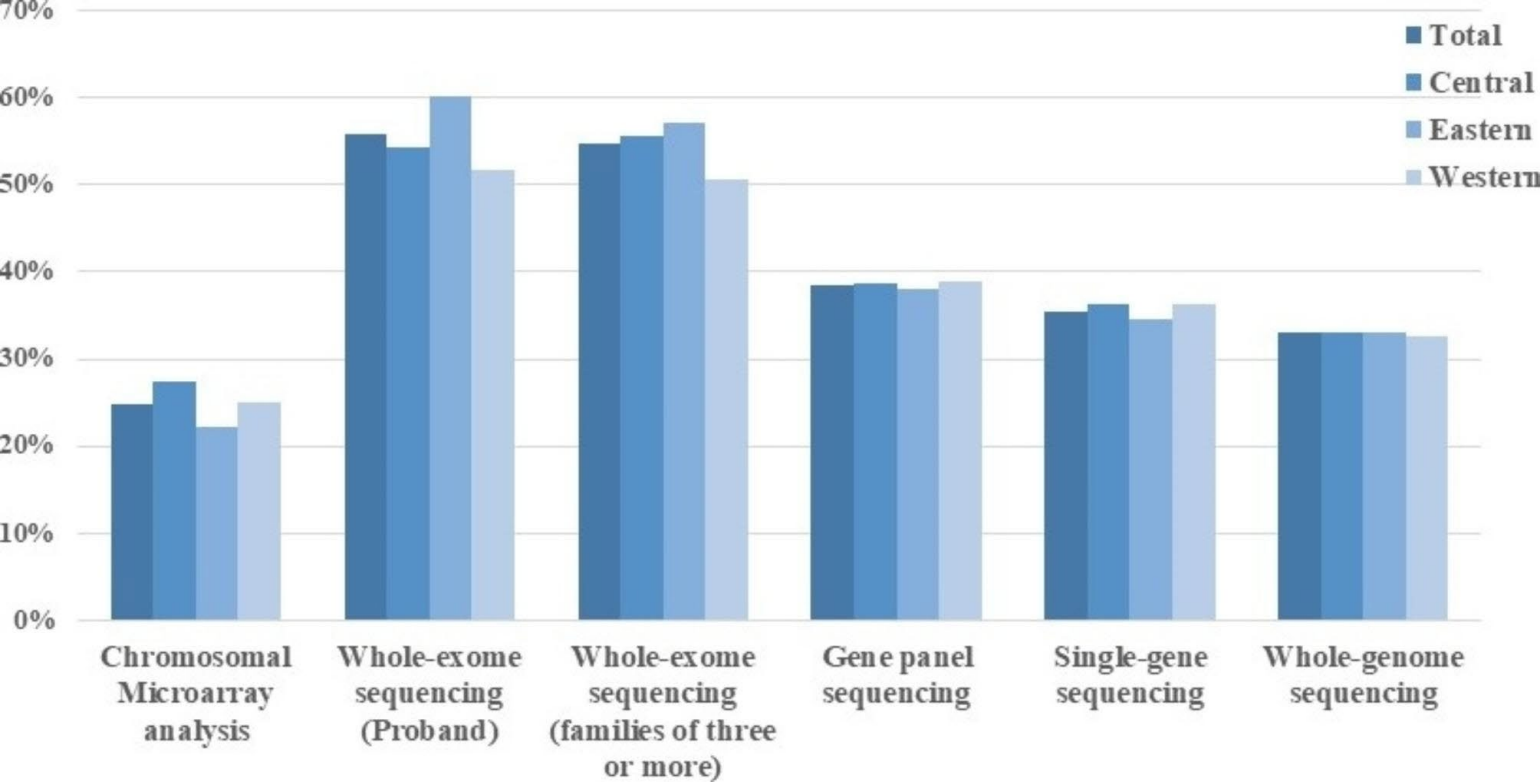
**Utilization rates of different genetic testing methods for rare diseases** (among 15,296 physicians who have used genetic testing for rare diseases)

Of the 15,296 physicians who used genetic testing to manage rare diseases, more than half used “Whole-exome sequencing (Proband)” and “Whole-exome sequencing (families of three or more) testing techniques. “Chromosomal Microarray analysis” was used by relatively few physicians (Fig. 2, Table S1). There were geographical differences (p < 0.001) in the use of Chromosomal Microarray analysis, Whole-exome sequencing (Proband), and Whole-exome sequencing (families of three or more) in rare diseases.

**Fig. 3:**
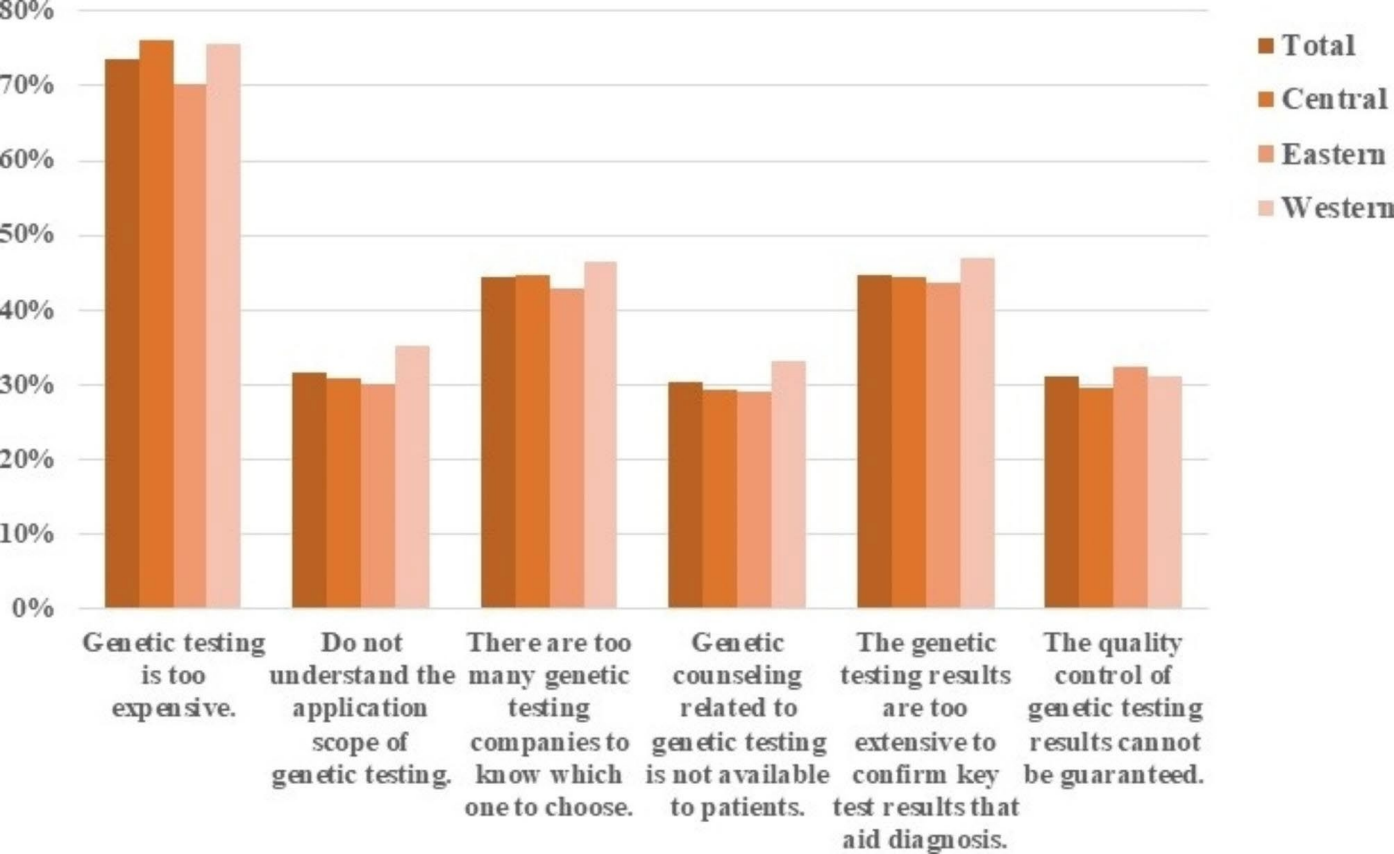
**Problems experienced by physicians in the use of genetic testing for rare diseases** (among 15,296 physicians who have used genetic testing for rare diseases)

In terms of genetic testing, the majority of doctors chose " too expensive” (73.6%), followed by “the report is too complicated to confirm the results” (44.7%) and " too many genetic companies on the market to know how to choose” (44.5%). Nearly 30% of the doctors were " unsure about the results of genetic testing”, “did not understand the scope of genetic testing” and “did not know how to advise patients on genetic aspects” (Fig. 3, Table S2).

## Discussion

This is the first cross-sectional study to describe the utilization rates of genetic testing in clinical practice for rare diseases. More than three-quarters of clinicians used genetic testing in rare disease care. The use of genetic testing was consistent with the level of economic development of each region, with the highest use of genetic testing in the most economically developed eastern region, followed by the central region, and the least in the western region. It can be roughly inferred that the accessibility of genetic testing varies across economic levels, which is similar to the findings of previous studies [21–23].

There is also a noticeable difference in the utilization rate of genetic testing for the diagnosis and treatment of rare diseases by physicians specializing in different fields, with over 90% of pediatricians using genetic testing for the diagnosis and treatment of rare diseases, compared to less than 60% of surgeons. This may be related to clinicians’ judgment on the examination required for disease diagnosis, and complex diseases in children may have a stronger genetic association [24, 25]. Access to genetic diagnosis for children is essential as it can lead to early and informed disease management and, in some cases, lifesaving interventions [26]. Previous studies have shown that 49% of children with neurodevelopmental disorders of unknown etiology report changes in clinical treatment or pathophysiological impressions after diagnosis by genetic testing [27]. The higher the education of doctors, the higher the utilization rate of genetic testing in the diagnosis and treatment of rare diseases. As a relatively new medical technology, genetic testing requires a high level of education from physicians.

Most physicians have used a variety of genetic tests in the management of rare diseases, the most popular methods were “Whole-exome sequencing (Proband)” and “Whole-exome sequencing (families of three or more)”. Whole-exome sequencing is the high-throughput sequencing of only 1% of the genome using sequence capture technology, detecting approximately 20,000 exons and 10 bp of introns upstream and downstream of the exons, and is the best option for patients with multiple symptoms, difficult diagnoses, and suspected genetic factors [28]. Compared with the testing of the proband only, the testing of family members can visualize the carrier status of each locus, and clarify whether the locus is de novo or inherited from the parents. For some autosomal recessive inherited diseases, we can determine whether it is in line with the genetic pattern according to the carrier status of the parents’ loci to avoid misdiagnosis [26]. It has also been reported that family testing is better in terms of positive rate [29]. The genetic testing methods used by clinicians currently vary, except for whole exome sequencing, which is the commonly used genetic test [30], and other methods chosen by physicians based on disease characteristics.

Physicians have encountered many problems with the use of genetic testing in the diagnosis and treatment of rare diseases, among which the high price was the main concern of medical workers. Although advances in technology have led to a reduction in the price of genetic tests, they are still expensive for the majority of patients compared to conventional diagnostics. Especially in most developing countries, genetic testing is not included in routine health insurance [31], and patients are required to pay the full cost of genetic testing. Given the necessity of genetic testing for the diagnosis and treatment of rare diseases, the government may consider providing certain safeguard policies to appropriately reduce the price of genetic testing and improve its accessibility. In addition, too many gene companies, opaque testing processes, non-standard test reports, and difficult-to-test results all made it challenging for clinicians to use genetic testing. This suggests that the government should strengthen the supervision and management of gene companies, strengthen quality control and standardize the reporting format of the genetic test, and assist with the interpretation of genomic data to enable the treating physician to make therapeutic and management decisions. At the same time, education and training on the scope of genetic testing and its interpretation should be carried out for medical specialties [32].

The main advantages of this study are that the sample size is relatively large, the survey scope is wide, and the findings cover different regions of China with different economic levels, which can provide relevant data support for the formulation of health policies. This study also has the following limitations. First of all, this study was only conducted in China, and the national policies in other countries should be considered when generalizing the results to other settings. Second, whether a doctor uses genetic testing technology in the diagnosis and treatment of rare diseases may be related to whether a rare disease is related to genetic factors. However, the field of rare diseases progresses slowly compared with common diseases, and it is not yet possible to determine that a particular rare disease is not related to genetic factors. And more and more diseases are finding breakthroughs at the genetic level. In addition, the experience with genetic testing for rare diseases is self-reported and cannot be verified. However, this is an inherent flaw of questionnaires. Moreover, our study was conducted with doctors, whose use of genetic testing is not driven by profit compared to other parties.

## Conclusions

In conclusion, our study showed that three-quarters of Chinese clinicians used genetic testing in the diagnosis and treatment of rare diseases, and there were regional differences in the utilization of genetic testing. Physicians have encountered many problems in the application of genetic testing technology in the diagnosis and treatment of rare diseases, with high prices being a major concern for medical workers. Our data contribute to identifying resource utilization patterns across different regions and providing a more comprehensive picture of the epidemiology of RDs in jurisdictions.

### Electronic supplementary material

Below is the link to the electronic supplementary material.

Supplementary Material 1: Utilization rate of different genetic testing methods and problems encountered in genetic testing

## Data Availability

The data that support the findings of this study are not openly available due to reasons of sensitivity and are available from the corresponding author upon reasonable request. Data are located in controlled access data storage at the State Key Laboratory of Complex Severe and Rare Diseases.

## References

[1] Haendel M, Vasilevsky N, Unni D, Bologa C, Harris N, Rehm H, Hamosh A, Baynam G, Groza T, McMurry J (2020). How many rare diseases are there?. Nat Rev Drug Discovery.

[2] The Lancet Diabetes E (2019). Spotlight on rare diseases. The lancet Diabetes & endocrinology.

[3] Gonzaludo N, Belmont JW, Gainullin VG, Taft RJ (2019). Estimating the burden and economic impact of pediatric genetic disease. Genet medicine: official J Am Coll Med Genet.

[4] Clark MM, Stark Z, Farnaes L, Tan TY, White SM, Dimmock D, Kingsmore SF (2018). Meta-analysis of the diagnostic and clinical utility of genome and exome sequencing and chromosomal microarray in children with suspected genetic diseases. NPJ genomic medicine.

[5] Nguyen MT, Charlebois K (2015). The clinical utility of whole-exome sequencing in the context of rare diseases - the changing tides of medical practice. Clin Genet.

[6] Stark Z, Tan TY, Chong B, Brett GR, Yap P, Walsh M, Yeung A, Peters H, Mordaunt D, Cowie S (2016). A prospective evaluation of whole-exome sequencing as a first-tier molecular test in infants with suspected monogenic disorders. Genet medicine: official J Am Coll Med Genet.

[7] Richardson R, Hingorani M, Van Heyningen V, Gregory-Evans C, Moosajee M. Clinical utility gene card for: Aniridia. Eur J Hum genetics: EJHG 2016, 24(11). 10.1038/ejhg.2016.73 PMC5110069 27381094

[8] Combs R, McAllister M, Payne K, Lowndes J, Devery S, Webster AR, Downes SM, Moore AT, Ramsden S, Black G (2013). Understanding the impact of genetic testing for inherited retinal dystrophy. Eur J Hum genetics: EJHG.

[9] Willig LK, Petrikin JE, Smith LD, Saunders CJ, Thiffault I, Miller NA, Soden SE, Cakici JA, Herd SM, Twist G (2015). Whole-genome sequencing for identification of mendelian disorders in critically ill infants: a retrospective analysis of diagnostic and clinical findings. The Lancet Respiratory medicine.

[10] Farnaes L, Hildreth A, Sweeney NM, Clark MM, Chowdhury S, Nahas S, Cakici JA, Benson W, Kaplan RH, Kronick R (2018). Rapid whole-genome sequencing decreases infant morbidity and cost of hospitalization. NPJ genomic medicine.

[11] Maiese DR, Keehn A, Lyon M, Flannery D, Watson M (2019). Current conditions in medical genetics practice. Genet medicine: official J Am Coll Med Genet.

[12] Fraiman YS, Wojcik MH (2021). The influence of social determinants of health on the genetic diagnostic odyssey: who remains undiagnosed, why, and to what effect?. Pediatr Res.

[13] Wojcik MH, Bresnahan M, Del Rosario MC, Ojeda MM, Kritzer A, Fraiman YS (2023). Rare diseases, common barriers: disparities in pediatric clinical genetics outcomes. Pediatr Res.

[14] Painous C, van Os NJH, Delamarre A, Michailoviene I, Marti MJ, van de Warrenburg BP, Meissner WG, Utkus A, Reinhard C, Graessner H (2020). Management of rare movement disorders in Europe: outcome of surveys of the european Reference Network for Rare Neurological Diseases. Eur J Neurol.

[15] Quinn L, Davis K, Yee A, Snyder H (2020). Understanding genetic learning needs of people affected by rare disease. J Genet Couns.

[16] Verberne EA, van den Heuvel LM, Ponson-Wever M, de Vroomen M, Manshande ME, Faries S, Ecury-Goossen GM, Henneman L, van Haelst MM (2022). Genetic diagnosis for rare diseases in the dutch Caribbean: a qualitative study on the experiences and associated needs of parents. Eur J Hum genetics: EJHG.

[17] Félix TM, Fischinger Moura de Souza C, Oliveira JB, Rico-Restrepo M, Zanoteli E, Zatz M, Giugliani R (2023). Challenges and recommendations to increasing the use of exome sequencing and whole genome sequencing for diagnosing rare diseases in Brazil: an expert perspective. Int J Equity Health.

[18] Liu P, Gong M, Li J, Baynam G, Zhu W, Zhu Y, Chen L, Gu W, Zhang S (2021). Innovation in Informatics to Improve Clinical Care and Drug Accessibility for Rare Diseases in China. Front Pharmacol.

[19] Guo J, Liu P, Chen L, Lv H, Li J, Yu W, Xu K, Zhu Y, Wu Z, Tian Z (2021). China’s first nation-wide rare diseases demographic analyses. Orphanet J Rare Dis.

[20] The National Health Commission. (2018). China’s First List of Rare Diseases. Available from: http://www.nhc.gov.cn/yzygj/s7659/201806/393a9a37f39c4b458d6e830f4 0a4bb9 9.shtml. 2019.

[21] Navarrete-Opazo AA, Singh M, Tisdale A, Cutillo CM, Garrison SR (2021). Can you hear us now? The impact of health-care utilization by rare disease patients in the United States. Genet medicine: official J Am Coll Med Genet.

[22] Chediak L, Bedlington N, Gadson A, Kent A, Khalek AA, Rosen L, Rust M, Shaikh MF, Tan MY, Wiafe SA (2022). Unlocking sociocultural and community factors for the global adoption of genomic medicine. Orphanet J Rare Dis.

[23] Robillard JM, Feng TL, Kabacińska K (2021). Access to genetic testing for rare diseases: existing gaps in public-facing information. World Med Health Policy.

[24] Smith HS, Franciskovich R, Lewis AM, Gerard A, Littlejohn RO, Nugent K, Rodriguez J, Streff H (2021). Outcomes of prior authorization requests for genetic testing in outpatient pediatric genetics clinics. Genet medicine: official J Am Coll Med Genet.

[25] Wright CF, FitzPatrick DR, Firth HV (2018). Paediatric genomics: diagnosing rare disease in children. Nat Rev Genet.

[26] Thomson KL, Ormondroyd E (2022). The genetic basis of primary Cardiomyopathies in Childhood: implications for clinical genetic testing. Circulation Genomic and precision medicine.

[27] Soden SE, Saunders CJ, Willig LK, Farrow EG, Smith LD, Petrikin JE, LePichon JB, Miller NA, Thiffault I, Dinwiddie DL (2014). Effectiveness of exome and genome sequencing guided by acuity of illness for diagnosis of neurodevelopmental disorders. Sci Transl Med.

[28] Yang Y, Muzny DM, Xia F, Niu Z, Person R, Ding Y, Ward P, Braxton A, Wang M, Buhay C (2014). Molecular findings among patients referred for clinical whole-exome sequencing. JAMA.

[29] Germain DP, Moiseev S, Suárez-Obando F, Al Ismaili F, Al Khawaja H, Altarescu G, Barreto FC, Haddoum F, Hadipour F, Maksimova I (2021). The benefits and challenges of family genetic testing in rare genetic diseases-lessons from fabry disease. Mol Genet Genom Med.

[30] Turro E, Astle WJ, Megy K, Gräf S, Greene D, Shamardina O, Allen HL, Sanchis-Juan A, Frontini M, Thys C (2020). Whole-genome sequencing of patients with rare diseases in a national health system. Nature.

[31] Riaz M, Tiller J, Ajmal M, Azam M, Qamar R, Lacaze P (2019). Implementation of public health genomics in Pakistan. Eur J Hum genetics: EJHG.

[32] Barwell J, Snape K, Wedderburn S. The new genomic medicine service and implications for patients *clinical medicine (London, England)* 2019, 19(4):273–7. 10.7861/clinmedicine.19-4-273 PMC6752257 31308102

