# Supplementary material for: Physicians’ use and perceptions of genetic testing for rare diseases in China: a nationwide cross-sectional study
Source: Orphanet J Rare Dis. 2023 Aug 10;18:240. doi: 10.1186/s13023-023-02847-7 (PMC10416371; doi:10.1186/s13023-023-02847-7)
Supplement: Supplementary file 1 — Supplementary Material 1: Utilization rate of different genetic testing methods and problems encountered in genetic testing [file 13023_2023_2847_MOESM1_ESM.docx]

**Appendix**

Table S1 Usage rates of different genetic testing methods for rare diseases (among physicians who have used genetic testing for rare diseases)

| Variables | Total | Central | Eastern | Western | P value |
| --- | --- | --- | --- | --- | --- |
|  | (N=15296) | (N=5421) | (N=5848) | (N=4027) |  |
| Chromosomal Microarray analysis | 3799 (24.8%) | 1493 (27.5%) | 1300 (22.2%) | 1006 (25.0%) | <0.001 |
| Whole-exome sequencing (Proband) | 8538 (55.8%) | 2939 (54.2%) | 3520 (60.2%) | 2079 (51.6%) | <0.001 |
| Whole-exome sequencing (families of three or more) | 8378 (54.8%) | 3010 (55.5%) | 3336 (57.0%) | 2032 (50.5%) | <0.001 |
| Gene panel sequencing | 5882 (38.5%) | 2091 (38.6%) | 2227 (38.1%) | 1564 (38.8%) | 0.731 |
| Single-gene sequencing | 5449 (35.6%) | 1967 (36.3%) | 2025 (34.6%) | 1457 (36.2%) | 0.128 |
| [Whole-genome sequencing](javascript:;) | 5044 (33.0%) | 1796 (33.1%) | 1937 (33.1%) | 1311 (32.6%) | 0.803 |

Table S2 Problems experienced by doctors in the use of genetic testing for rare diseases (among physicians who have used genetic testing for rare diseases)

| Variables | Total | Central | Eastern | Western | P value |
| --- | --- | --- | --- | --- | --- |
|  | (N=15296) | (N=5421) | (N=5848) | (N=4027) |  |
| Genetic testing is too expensive. | 11263 (73.6%) | 4122 (76.0%) | 4102 (70.1%) | 3039 (75.5%) | <0.001 |
| Do not understand the application scope of genetic testing. | 4834 (31.6%) | 1668 (30.8%) | 1752 (30.0%) | 1414 (35.1%) | <0.001 |
| There are too many genetic testing companies to know which one to choose. | 6800 (44.5%) | 2427 (44.8%) | 2504 (42.8%) | 1869 (46.4%) | 0.002 |
| Genetic counseling related to genetic testing is not available to patients. | 4634 (30.3%) | 1588 (29.3%) | 1709 (29.2%) | 1337 (33.2%) | <0.001 |
| The genetic testing results are too extensive to confirm key test results that aid diagnosis. | 6842 (44.7%) | 2407 (44.4%) | 2548 (43.6%) | 1887 (46.9%) | 0.005 |
| The quality control of genetic testing results cannot be guaranteed. | 4750 (31.1%) | 1607 (29.6%) | 1887 (32.3%) | 1256 (31.2%) | 0.011 |
